# Diet-induced obesity accelerates oral carcinogenesis by recruitment and functional enhancement of myeloid-derived suppressor cells

**DOI:** 10.1038/s41419-021-04217-2

**Published:** 2021-10-14

**Authors:** Jianmin Peng, Qinchao Hu, Xijuan Chen, Chunyang Wang, Jiayu Zhang, Xianyue Ren, Yun Wang, Xiaoan Tao, Huan Li, Ming Song, Bin Cheng, Tong Wu, Juan Xia

**Affiliations:** 1 Hospital of Stomatology, Sun Yat-sen University, Guangzhou, China; 2 Guangdong Provincial Key Laboratory of Stomatology, Guangzhou, China; 3 Guanghua School of Stomatology, Sun Yat-sen University, Guangzhou, China; 4 State Key Laboratory of Oncology in South China, Collaborative Innovation Center of Cancer Medicine, Guangzhou, China; 5 Department of Intensive Care Unit, Sun Yat-sen University Cancer Center, Guangzhou, China; 6 Department of Head and Neck Surgery, Sun Yat‑sen University Cancer Center, Guangzhou, China

**Keywords:** Cancer microenvironment, Oral cancer

## Abstract

Although obesity has been associated with an increased risk and aggressiveness of many types of carcinoma, whether it promotes squamous cell carcinoma remains unclear. To reveal the role of obesity in oral squamous cell carcinoma (OSCC) initiation and development, we used 4NQO-induced OSCC model mice to examine the impact of dietary obesity on carcinogenesis. The results showed that high-fat diet (HFD)-induced obesity significantly promoted the incidence of OSCC and altered the local immune microenvironment with the expansion of CD11b^+^Gr1^+^ myeloid-derived suppressor cells (MDSCs). The underlying mechanism that induced an immunosuppressive local microenvironment in obesity was the recruitment of MDSCs through the CCL9/CCR1 axis and enhancement of MDSC immunosuppressive function via intracellular fatty acid uptake. Furthermore, clinical samples verified the increase in infiltrated CD33^+^ (a marker of human MDSCs) cells in obese OSCC patients, and data from the TCGA dataset confirmed that CD33 expression was positively correlated with local adipocytes in OSCC. Survival analysis showed that enrichment of adipocytes and high expression of CD33 were associated with poor prognosis in OSCC patients. Strikingly, depletion of MDSCs significantly ameliorated HFD-promoted carcinogenesis in 4NQO-induced model mice. These findings indicate that obesity is also an important risk factor for OSCC, and cancer immunotherapy, especially targeting MDSCs, may exhibit greater antitumor efficacy in obese patients.

## Introduction

Obesity is a serious worldwide health problem attracting public concern. According to the World Health Organization (WHO), the incidence of obesity in adults has nearly tripled from 1975 to 2016, with over 650 million people affected [1, 2]. In addition to type 2 diabetes and other traditional comorbidities, the association between obesity and several cancers, such as breast, kidney (renal cell), colon, pancreas, gallbladder, and liver cancer, has been well established [3]. However, whether obesity promotes the development of squamous cell carcinoma remains unclear. In a previous study, we found that obesity was associated with poor prognosis in early-stage (T1/2N0M0) oral squamous cell carcinoma (OSCC) patients [4]. More studies are needed to further identify the relationship between obesity and epithelial carcinogenesis, as well as the underlying pathophysiological mechanisms. Understanding how obesity impacts the initiation and development of OSCC may result in improved early intervention strategies to decrease oral cancer risk.

Obesity has been reported to promote tumorigenesis through a variety of mechanisms, among which a chronic inflammatory state and immune response dysregulation are of vital importance [5, 6]. Adipose tissue is regarded as an active endocrine organ that secretes a series of inflammatory mediators such as IL-5 and GM-CSF, which cause myeloid cells to differentiate and stimulate their migration to the local tissue. These complex components form a premetastatic microenvironment that is conducive to tumor growth [7, 8]. Moreover, as obesity is associated with excess lipids, the effect of obesity on lipid metabolism in immune cells has attracted much attention [9]. It has been reported that NK cell uptake of lipids from the environment in obesity activates the PPARα/δ pathways, causing “cellular metabolic paralysis” and inducing antitumor function defects [10]. Taken together, obesity may remodel the components and function of immune cells to establish an immunosuppressive microenvironment and thereby promote the initiation and development of squamous cell carcinoma.

Myeloid-derived suppressor cells (MDSCs) are immune-suppressive cells that play essential roles in facilitating tumor growth [11]. They exert immunosuppressive functions by preventing the activation of T cells, inhibiting the toxic effect of NK cells, and inducing regulatory T cells [12]. The number of MDSCs is very small in healthy individuals but is amplified under disease conditions. Chronic low-grade inflammatory and intracellular lipid accumulation were reported to impact the number and function of MDSCs [13, 14]. Therefore, we hypothesized that obesity, characterized by a chronic inflammatory state, may regulate the number and function of MDSCs to generate an immunosuppressive microenvironment, thereby participating in the carcinogenesis process.

Here, we first used wild-type C57BL/6 mice to examine the impact of dietary obesity on the initiation and development of OSCC. We found that high-fat diet (HFD)-induced obesity significantly promoted oral carcinogenesis with the expansion of MDSCs in the local microenvironment. Further study revealed that the recruitment of MDSCs was mediated by the CCL9/CCR1 axis, and HFD enhanced the suppressive potency of MDSCs by increasing the intracellular uptake of lipids. Strikingly, depletion of MDSCs strongly suppressed the development of OSCC and was particularly effective in obese conditions.

## Materials and methods

### Animals, cells, and clinical samples

Four-week-old male wild-type C57BL/6 mice were purchased from GemPharmatech Co. LTD and housed in the Experimental Animal Center at Sun Yat-sen University North Campus. Mice were fed either a high-fat diet (HFD, 60% kcal, D12492) or a matched normal-fat diet (NFD, 10% kcal, D12450B). SCC7 murine squamous cell carcinoma cells were cultured in DMEM supplemented with 10% FBS. Primary tongue epithelial cells were isolated from mice as described in previous studies [15]. Briefly, the tongue was washed and sterilized with PBS containing 2% penicillin-streptomycin (Life Technologies, USA) and then cut into small pieces. After that, tissues were digested using 1 mg/mL collagenase IV (Gibco, USA) for 30 min followed by 0.25% trypsin-EDTA (Gibco, USA) for 5 min to produce single-cell suspension. Primary cells were cultured in K-SFM medium (Gibco, USA) specific for epithelial cells. Paraffin-embedded clinical samples of OSCC were collected from Sun Yat-sen University Cancer Center. To avoid the impact of prediagnosis weight loss in late-stage patients, only T1/2N0M0 cases were collected. TNM stages were classified according to the 7th edition of the AJCC cancer staging manual [16]. According to body mass index (BMI), the cases were categorized as obese (BMI ≥ 28 kg/m^2^) or nonobese (18.5 kg/m^2^ kg < BMI < 28 kg/m^2^) [4]. In total, 20 obese OSCC samples and 22 nonobese OSCC samples were obtained.

### Chemical reagents and antibodies

The carcinogen 4-nitroquinoline-1-oxide (4NQO) was purchased from Sigma–Aldrich and diluted in sterile water to a final concentration of 50 μg/mL or 100 μg/mL. The in vivo anti-mouse Ly6G/Ly6C (Gr1) neutralizing antibody was obtained from BioXcell. Palmitic acid (PA), oleic acid (OA), and oil red O were provided by Sigma–Aldrich. DNase I was acquired from Biofroxx. FcR-Blocking Reagent (anti-mouse CD16/32), Zombie Green^TM^ Fixable Viability Kit and the following fluorescently labeled antibodies were from BioLegend: anti-mouse mAbs CD45-BV605, Ly6G/Ly6C (Gr1)-BV510, Gr1-AF594, Ly6C-BV421, Ly6G-PE, CD3-APC/Cy7, CD4-PE/Cy7, CD8a- PerCP/Cy5.5, CCR1 (CD191)-APC, CD11b-APC, and CD11b-AF700. RBC lysis buffer, IC fixation buffer, permeabilization buffer (10×), and anti-mouse mAbs Arg1-APC and iNOS-PE were obtained from eBioscience. The CellTrace™ Violet Cell Proliferation Kit, BODIPY^TM^ 493/503, and immunostaining antibody against CCR1 were from Life Technologies. Rabbit anti-mouse Ki67 antibody was purchased from Arigo Biolaboratories. Primary anti-mouse/human CD11b, GAPDH, anti-human CD33, and anti-mouse MIP1γ (CCL9) antibodies were purchased from Abcam. Immunostaining antibody against Ly6G was acquired from Servicebio. CCL9 protein was from NovoProtein. Secondary antibodies coupled to fluorescent Alexa probes were purchased from Beijing Emarbio Science Technology.

### Experimental animal model and MDSC depletion

**Fig. 1:**
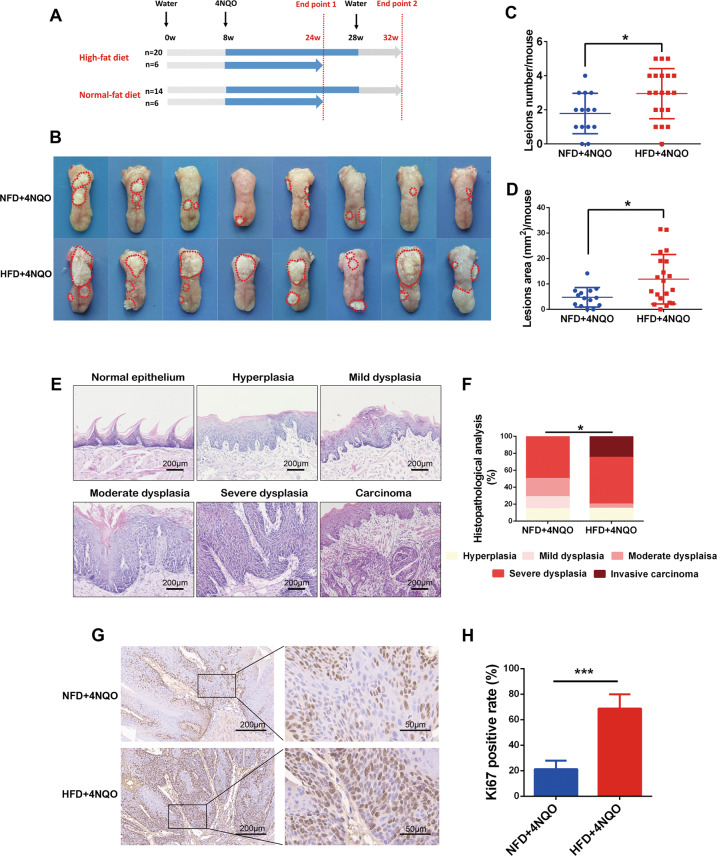
HFD accelerated oral carcinogenesis in a 4NQO-induced mouse model. **A** Schematic diagram of animal model construction. 4NQO concentration: 50 μg/mL. **B** Representative gross observation of the tongues in different groups at endpoint 2. **C**, **D** Quantification of the lesion number and lesion size in different groups at endpoint 2. **E** Representative H&E staining of different pathological grades, including normal epithelium, hyperplasia, mild dysplasia, moderate dysplasia, severe dysplasia, and carcinoma. **F** Quantification of pathological grade in different groups at endpoint 2. **G**, **H** Representative images and quantification of immunohistochemistry staining of Ki67 for the lesions (severe dysplasia) in different groups. Single asterisk (*) indicates *P* < 0.05, double asterisks (**) indicate *P* < 0.01, triple asterisks (***) indicate *P* < 0.001. Unpaired *t*-test; Mann–Whitney test. NFD normal-fat diet, HFD high-fat diet.

Mice were randomly divided into the HFD and NFD groups. After 8 weeks of feeding, 4NQO was added to the drinking water to induce carcinogenesis in the tongue, and the diets were maintained. To better assess the cancer-promoting effect of a HFD, a relatively low concentration of 4NQO (50 μg/mL) was used. All animals underwent a biweekly full oral cavity examination under anesthesia. Before tumorigenesis, mice were randomly selected from each group and sacrificed after exposure to carcinogen for 16 weeks (the first endpoint). The other mice were kept on 4NQO drinking water for the indicated time and then were reverted to regular water and monitored until the end of the experiment (Fig. 1A).

For MDSC depletion, mice fed a NFD/HFD were provided with 4NQO (100 μg/mL) for 16 weeks, followed by IP injection of Gr1 mAb (6 µg g^−1^ body weight anti-Gr1 dosed every 3 days, clone RB6-8C5) or isotype-matched control IgG.

### Sample collection and processing

Mice were euthanized at the indicated time points for tissue retrieval. Blood, tongue, spleen, liver, and bone marrow from femurs and tibias were collected. Tongue lesions were photographed and lesion area was measured using CADSee software (CADSee V8.2.0, China). Then, the tongue was sectioned longitudinally from the center of the lesions. One half was fixed and processed with paraffin embedding for further histopathologic diagnosis and immunohistochemical studies. The other half of the tissue at the first endpoint (before tumorigenesis) was snap-frozen in liquid nitrogen for microarray assays. The other half of the tissue at the second endpoint was frozen for further study or processed into a single-cell suspension for flow analysis. Briefly, tissue was dissected, minced, digested with enzymes (1 mg/mL collagenase IV and 0.1 mg/mL DNase I) at 37 °C for 30 min and passed through a 70 μm strainer to obtain a single-cell suspension. The spleen and bone marrow were ground and incubated with RBC lysis buffer to remove red cells. For immune cell acquisition, blood was anticoagulated with heparin and incubated with lysis buffer. To extract serum for lipid detection, blood was procoagulated for 30 min. The liver was processed into OCT-embedded frozen sections for oil red O staining.

### Lipid, weight, and blood glucose detection

Mice were fasted for 8 h, and then their serum was harvested for total cholesterol (TC) and triglyceride (TG) analysis. For lipid detection, frozen liver sections were fixed in ice-cold acetone and stained in 0.3% oil red O for 10 min, followed by 70% ethanol to wash away the remaining dye. After washing with distilled water, slides were counterstained with hematoxylin for 10 s. Body weight was recorded every 2 weeks. Fasted blood glucose was measured using a glucose meter (LifeScan).

### Histopathological diagnosis and immunostaining

Paraffin-embedded sections of the tongue were used for hematoxylin and eosin (H&E) staining for histopathology. Histopathological diagnosis was performed by an experienced oral pathologist according to the pathological grading criteria of epithelial dysplasia described by the WHO in 2006 [17]. For immunohistochemical and immunofluorescence analysis, tissue sections were processed as previously described [18]. Briefly, 5 μm tongue sections were deparaffinized and rehydrated in a series of gradient alcohols. Then, slides were immersed in sodium citrate buffer and boiled in a microwave for antigen retrieval. After inhibition of endogenous peroxidases and blocking, samples were covered with primary antibodies and incubated at 4 °C overnight. The following antibodies were used: Ki67, Ly6G, CD33, CCR1, CD11b, and Gr1 conjugated with Alexa Fluor 594. The next day, HRP-conjugated secondary antibodies were applied for 30 min and visualized with DAB, or secondary Alexa Fluor 488 dye-conjugated antibodies were applied for 30 min at room temperature. Nuclear counterstaining was performed using hematoxylin or 4’,6-diamidino-2-phenylindole (DAPI). The positive rate of Ki67 in lesions were calculated as the following: (number of nuclear positive epithelial cells/number of total epithelial cells) ×100%. At least five fields (magnification ×400) were counted per section and the average positive rates were calculated [19]. The stained cells were calculated using Image J software (National Institutes of Health, USA).

### Flow cytometry analysis

Single-cell suspensions of blood, spleen, or tongue tissue obtained as described above were incubated with Fc receptor-blocking antibody before staining. Cells were processed for live/dead cell discrimination using a Zombie Green^TM^ Fixable Viability Kit and then stained for 30 min on ice with the following fluorochrome-conjugated antibodies: CD45, CD11b, CD3, Gr1, Ly6G, Ly6C, and CCR1. Subsequently, fixation and permeabilization buffer was applied for intracellular staining. For iNOS and Arg1 production evaluation, cells were incubated with antibodies for 4 h 4 °C. Assessment of intracellular lipids was performed by incubation of cells with 1 μM BODIPY 493/503 at 37 °C for 30 min as recommended by the manufacturer. Stained cells were analyzed on a flow cytometer (CytExpert, Beckman Coulter).

### Antibody array

To detect cytokines in the tongue of HFD- and NFD-fed mice, whole protein samples were isolated from frozen tissue at the first endpoint and applied to the Mouse Cytokine Array 6 (AAMCYT-G6, RayBiotech). The detailed experimental procedure was developed according to the manufacturer’s instructions. Then, the films were scanned using a chemiluminescence imaging system and the intensity of the signals was analyzed by ImageJ.

### Quantitative real-time polymerase chain reaction (qPCR)

Total RNA was extracted from frozen tongue tissue by TRIzol (Invitrogen, Carlsbad, CA, USA) and reverse transcribed by a High Capacity cDNA Synthesis Kit (Roche, Mannheim, Germany). Real-time quantitative RT–PCR (SYBR Green, Roche) was performed to quantify mRNA levels. GAPDH was used as the reference gene. The following primers were used:

GAPDH forward 5’-AGGTCGGTGTGAACGGATTTG-3’

GAPDH reverse 5’-TGTAGACCATGTAGTTGAGGTCA-3’

S100A9 forward 5’-GCACAGTTGGCAACCTTTATG-3’

S100A9 reverse 5’-TGATTGTCCTGGTTTGTGTCC-3’

ARG1 forward 5’-CTCCAAGCCAAAGTCCTTAGAG-3’

ARG1 reverse 5’-GGAGCTGTCATTAGGGACATCA-3’

iNOS forward 5’-GGGCTGTCACGGAGATCA-3’

iNOS reverse 5’-CCATGATGGTCACATTCTGC-3’

### Western blotting (WB)

Adipose tissue isolated from male wild-type C57BL/6 mice was minced thoroughly into pieces and incubated for 24 h to generate conditioned media (CM). Primary tongue epithelial cells and SCC7 cells were treated with adipocyte-CM, palmitic acid, or oleic acid for 48 h. To obtain total protein, cells and frozen tissue were lysed with RIPA buffer (Sigma–Aldrich) supplemented with protease and phosphatase inhibitors. Equal amounts of protein extracts were separated by SDS-PAGE and transferred to PVDF membranes (Millipore, MA, USA). After blocking with nonfat milk, the membranes were incubated with primary antibodies (CCL9, GAPDH) overnight at 4 °C followed by a secondary antibody. Signals were detected using enhanced chemiluminescence (ECL) (Millipore).

### ELISA

After treatment with adipocyte-CM and fatty acids, the cell supernatant was discarded and replaced with fresh medium. Cells were incubated at 37 °C for 24 h. To detect CCL9 secreted by SCC7 and primary tongue epithelial cells, medium from different groups was collected and subjected to ELISA analysis. The mouse CCL9 ELISA kit was purchased from Boster (Wuhan, China). The experimental procedure was performed according to the manufacturer’s recommendation.

### MDSC isolation and chemotaxis assays

MDSCs were generated as previously described [20]. Bone marrow cells of healthy C57BL/6 mice were stimulated with 20 ng/mL GM-CSF and 10 ng/mL IL-4 for 3 days. Nonadherent cells were collected for staining. After excluding dead cells, CD11b^+^Gr1^+^ cells were sorted by FACS for subsequent study. CD11b^+^Gr1^+^ cells isolated from the spleens of diet-induced mice (fed a NFD or HFD for 8 weeks) were subjected to intracellular lipid detection and immunosuppressive activity evaluation.

Chemotaxis assays of murine MDSCs were performed using 24-well plates with Transwell inserts (5.0 μm; Corning) with some modifications [21]. Briefly, 1 × 10^6^ BM-derived MDSCs were seeded in the upper chambers with serum-free medium. The lower chamber contained complete medium with or without CCL9 (10 ng/mL, 50 ng/mL, or 100 ng/mL). After incubation for 24 h, the number of migrated cells was counted.

### In vitro suppression assays

Peripheral blood mononuclear cells (PBMCs) from healthy mice were labeled with CellTrace™ violet fluorescence according to the manufacturer’s instructions. Subsequently, labeled cells (1 × 10^5^ cells/well) were cocultured with MDSCs (pretreated with PA or OA, isolated from HFD or NFD mice) at different ratios in the presence of 1 μg/ml plate-bound anti-CD3 (145-2C11, BioLegend) and 2 μg/ml anti-CD28 (37.51, BioLegend). After 3 days of incubation, cells were harvested and subsequently stained for CD3, CD4, and CD8 cell surface markers. The proliferation of T cells was determined based on CellTrace™ violet fluorescence dilution using flow cytometry.

### The Cancer Genome Atlas (TCGA) database analysis

The mRNA sequencing data and clinical information were downloaded from the TCGA data portal. A total of 331 OSCC cases with both sequencing data and corresponding clinical information were obtained. Estimation of the abundance of adipocytes was performed by the xCell algorithm [22], and the data were downloaded from https://xcell.ucsf.edu/. Correlation analysis was performed by Pearson regression. Receiver operating characteristic (ROC) curves were used to determine the optimal cutoff, with the highest score of both sensitivity and specificity, to divide patients into high expression or low expression groups. Survival curves were plotted by the Kaplan–Meier method and compared by the log-rank test.

### Statistical analysis

Statistical analysis was performed with SPSS 23.0 software (IBM, Armonk, USA) and GraphPad Prism 5.0 (GraphPad Software, USA). Sample sizes and replicates were chosen according to previous studies and are indicated in each method described above. The quantitative data are presented as the mean ± standard deviation (SD). The Mann–Whitney test, Kruskal–Wallis test, Student’s *t*-test, and one-way ANOVA were used to compare the differences among variants. A two-tailed *P* value < 0.05 was considered statistically significant.

## Results

### Diet-induced obesity accelerated oral carcinogenesis in a 4NQO-induced mouse model

To elucidate whether diet-induced obesity promotes the development of OSCC, C57BL/6 mice were exposed to either a NFD or a HFD for 8 weeks, and then the NFD- and HFD-fed mice were subjected to the establishment of oral carcinogenesis induced by drinking 4NQO water. A schematic diagram of animal model construction is shown in Fig. 1A. As expected, animals exhibited an obesity phenotype with increased body weight, serum lipid contents (TG and TC), and blood glucose from the eighth week of HFD feeding (Supplementary Fig. S1A–E). In addition, we evaluated the fat contents in local tissues. After 24 weeks of feeding, the HFD-fed mice developed fatty liver, with a large number of lipid droplets in the liver cells (Supplementary Fig. S1F, G). Remarkably, fat cells accumulated under the epithelium of the tongue in HFD-fed mice (Supplementary Fig. S1H, I).

In the precancer phase (16 weeks of 4NQO exposure), visible white patches appeared on the tongue. The pathological manifestations were dysplasia with an intact epithelial basement membrane (Supplementary Fig. S2A). Strikingly, more immune cells accumulated in dysplastic lesions in the HFD + 4NQO group than in the NFD + 4NQO group Supplementary Fig. S2B), suggesting that HFD may have effects on the immune microenvironment during carcinogenesis.

After 4NQO exposure for 20 weeks, the lesions were obvious at this stage, showing white verrucous, cauliflower-like, granular hyperplasia and white flakes and spot plaques. The lesions in the HFD + 4NQO group were accompanied by erosion, ulcers, and endogenous growth with unclear boundaries (Fig. 1B). The average lesion number (2.95 ± 0.328 vs. 1.79 ± 0.318, *P* < 0.05) and lesion size (9.08 ± 1.09 vs. 4.64 ± 1.05, *P* < 0.05) were significantly higher in the HFD + 4NQO group than in the NFD + 4NQO group (Fig. 1C, D). Histopathologic evaluation showed that different stages of pathogenesis varied from normal epithelium to hyperplasia, mild dysplasia, moderate dysplasia, severe dysplasia, and carcinoma (Fig. 1E), according to the pathological grading criteria described by the WHO in 2006 [17]. As summarized in Fig. 1F, a total of 25% (5/20) of mice in the HFD + 4NQO group developed OSCC, while no invasive carcinoma was found in the NFD + 4NQO group. The disease severity between the two groups was compared, and the results showed that the HFD + 4NQO group had a significantly elevated severity of oral carcinogenesis (Fig. 1F). Moreover, the proliferative capacity of the dysplastic lesions was evaluated by IHC staining with Ki67. The positive rate of Ki67 in the HFD + 4NQO mice was significantly higher than that in the NFD + 4NQO mice (Fig. 1G, H). These results indicated that diet-induced obesity promoted oral carcinogenesis in a 4NQO mouse model.

### HFD caused immune microenvironment changes and promoted CD11b^+^Gr1^+^ MDSC accumulation in the tongue of mice

**Fig. 2:**
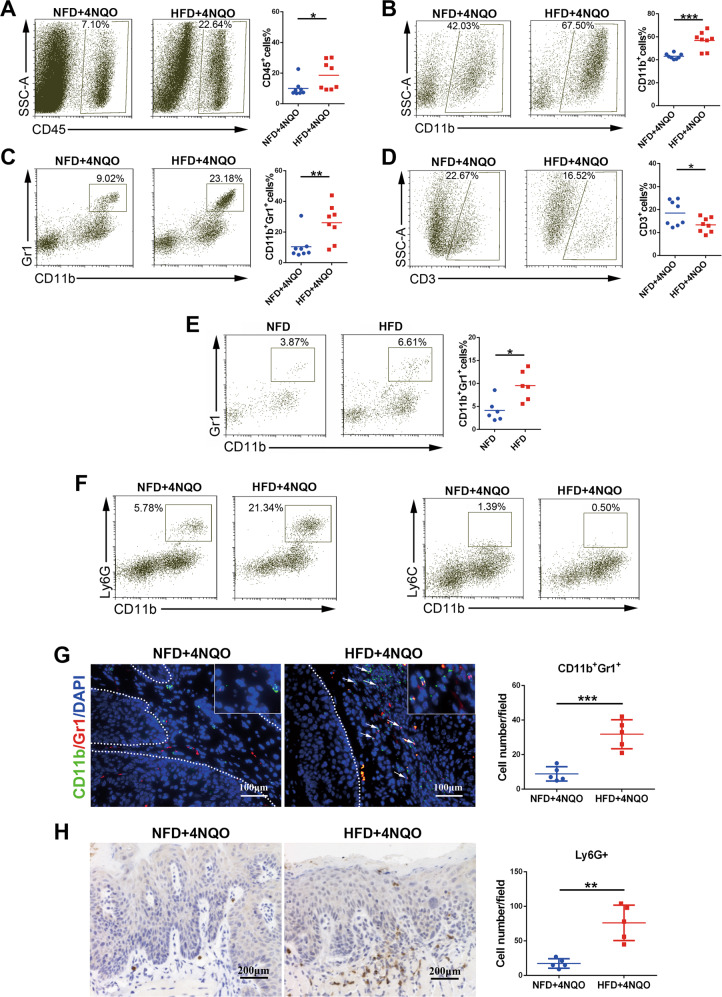
HFD changed the immune microenvironment and promoted CD11b^+^Gr1^+^ MDSC accumulation in the tongue of mice. **A–D** Representative dot plot and quantification of CD45^+^ immune cells (**A**), CD11b^+^ myeloid cells (**B**), CD11b^+^Gr1^+^ MDSCs (**C**), and CD3^+^ T cells (**D**) in the tongues of NFD + 4NQO or HFD + 4NQO mice by flow cytometry. **E** Representative dot plot and quantification of CD11b^+^Gr1^+^ MDSCs in the tongues of NFD or HFD mice (without 4NQO exposure). **F** Representative dot plot of polymorphonuclear MDSCs (Ly6G) and monocytic MDSCs (Ly6C) in the tongues of NFD + 4NQO or HFD + 4NQO mice. **G** Representative images and quantification of immunofluorescence staining of CD11b^+^Gr1^+^ MDSCs in the tongues of NFD + 4NQO or HFD + 4NQO mice. **H** Representative images and quantification of immunohistochemical staining of LY6G in the tongues of NFD + 4NQO or HFD + 4NQO mice. Single asterisk (*) indicates *P* < 0.05, double asterisks (**) indicate *P* < 0.01, triple asterisks (***) indicate *P* < 0.001. Unpaired *t*-test. NFD normal-fat diet, HFD high-fat diet, MDSCs myeloid-derived suppressor cells.

As immune cell alteration was observed in the precancer phase, we further investigated the effect of a HFD on the local immune microenvironment at the end of the experiment. The immunocyte profiles in the tongue of model mice were analyzed by flow cytometry (gating strategy in Supplementary Fig. S3). We found that HFD + 4NQO tongues exhibited elevated proportions of CD45^+^ leukocytes (Fig. 2A), most of which were CD11b^+^ myeloid cells (Fig. 2B). Moreover, the proportion of CD11b^+^Gr1^+^ MDSCs in the HFD + 4NQO group was significantly higher than that in the NFD + 4NQO group (26.09 ± 4.23% vs. 10.43 ± 2.97%, Fig. 2C), while the proportion of CD3^+^ T lymphocytes was lower in the HFD + 4NQO group (Fig. 2D). In addition, we detected alterations in MDSCs in the tongues of mice after 8 weeks of HFD feeding without 4NQO exposure. The results also showed that the proportion of CD11b^+^Gr1^+^ cells was higher in the HFD group than in the NFD group (Fig. 2E), suggesting that a high-fat diet induced an immunosuppressive microenvironment by expanding MDSCs. MDSCs in local tissues usually migrate from the circulatory system; thus, we further analyzed the proportion of MDSCs in peripheral circulating blood and in the spleen. The results indicated that HFD feeding did not affect the MDSC populations in the spleen in either condition with or without 4NQO exposure (Supplementary Fig. S4A, C); however, the proportion of MDSCs was elevated in the blood of the HFD and HFD + 4NQO groups compared with the control groups (Supplementary Fig. S4B, D), which may favor the migration of MDSCs into local organs in HFD-fed mice. MDSCs are a heterogeneous group of cells that are further divided into two subsets: monocytic MDSCs (M-MDSCs, CD11b^+^Ly6G^-^Ly6C^high^) and polymorphonuclear MDSCs (PMN-MDSCs, CD11b^+^Ly6G^+^Ly6C^low^). We found that the myeloid cells in the tongue mainly expressed Ly6G (Fig. 2F), indicating that the predominant subpopulation of local MDSCs was PMN-MDSCs. Furthermore, immunofluorescence and immunohistochemical experiments were used to verify the in situ infiltration of MDSCs (CD11b^+^Gr1^+^) and their subtype (Ly6G^+^) cells in lesions. As expected, more CD11b^+^Gr1^+^ cells (Fig. 2G) and Ly6G+ cells (Fig. 2H) infiltrated in the dysplastic lesion in the HFD + 4NQO group than in the NFD + 4NQO group. These results suggested that a HFD may accelerate the process of oral carcinogenesis by accumulating local MDSCs, especially PMN-MDSCs.

### MDSCs were recruited to the oral microenvironment through the CCL9/CCR1 axis in HFD mice

**Fig. 3:**
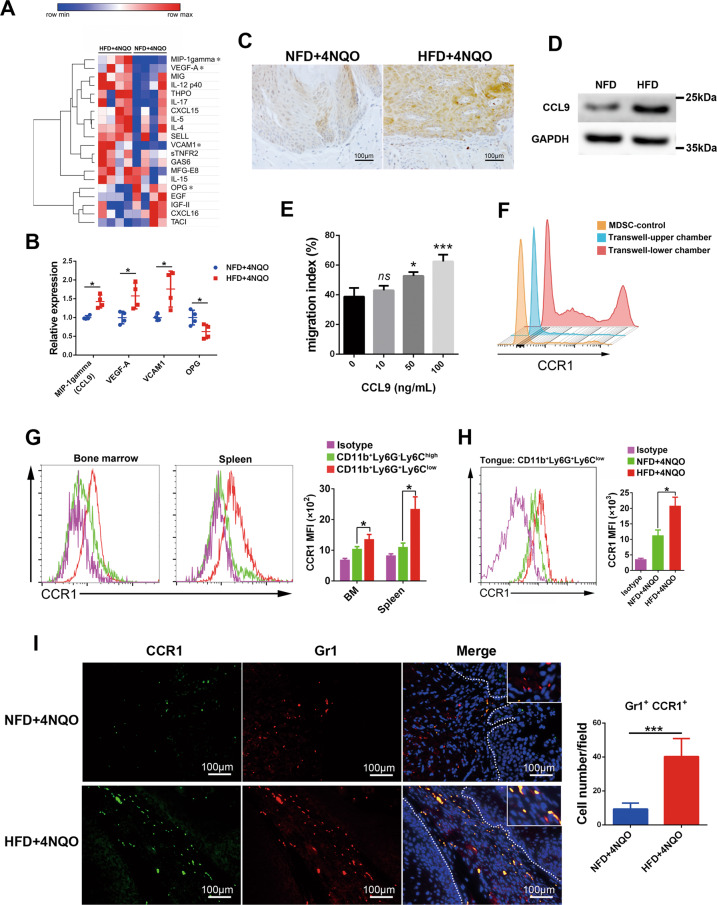
MDSCs were recruited to the oral microenvironment through the CCL9/CCR1 axis in HFD mice. **A, B** Heatmap and quantification of the antibody arrays in tongues from NFD + 4NQO and HFD + 4NQO mice at the precancer stage (endpoint 1). **C** Representative images of immunohistochemical staining of CCL9 in tongue lesions. **D** Western blot analysis of the expression of CCL9 in the tongues of NFD or HFD mice (without 4NQO exposure). **E** Transwell assays showed the ability of CCL9 to promote the migration of MDSCs. MDSCs were seeded in the upper chamber, while different concentrations of CCL9 were added to the lower chamber. **F** When 50 ng/mL CCL9 was added, the expression of CCR1 on MDSCs that migrated into the lower chamber or remained in the upper chamber was analyzed by flow cytometry. **G** Flow cytometry analysis showed the expression of CCR1 on PMN-MDSCs (CD11b^+^Ly6G^+^Ly6^low^) and M-MDSCs (CD11b^+^Ly6G^-^Ly6^high^) from bone marrow or spleen. **H** Flow cytometry analysis showed the expression of CCR1 on PMN-MDSCs from the tongues of NFD + 4NQO or HFD + 4NQO mice. **I** Representative images of immunofluorescence staining of CCR1 and Gr1 in dysplastic lesions in NFD + 4NQO or HFD + 4NQO mice. Single asterisk (*) indicates *P* < 0.05, triple asterisks (***) indicate *P* < 0.001. Unpaired *t*-test. NFD normal-fat diet, HFD high-fat diet, MDSCs myeloid-derived suppressor cells, PMN-MDSCs polymorphonuclear MDSCs, M-MDSCs monocytic MDSCs.

The migration and accumulation of local MDSCs are usually regulated by chemotactic cytokines. To clarify the mechanisms of MDSC enrichment in the tongue of model mice, an antibody array was adopted to detect cytokines in the precancer stage. The expression of CCL9, VEGF-A, and VCAM1 were highly expressed in the HFD + 4NQO group, while OPG was expressed at low level in the HFD + 4NQO group (Fig. 3A, B). CCL9, also known as macrophage inflammatory protein-1 gamma (MIP1γ), is often expressed in macrophages, osteoclasts, and epithelial cells of mice and has been reported as a chemokine that recruits myeloid cells. To verify the effect of a HFD on the production of CCL9 and its localization in tissues, immunohistochemical analysis of CCL9 expression was performed. CCL9 was mainly expressed on epithelial cells, and the expression level of the HFD + 4NQO group was higher than that of the NFD + 4NQO group (Fig. 3C). In addition, western blot results showed the upregulation of CCL9 in the tongue of HFD-fed mice without carcinogen induction, confirming that the HFD induced the production of CCL9 in the tongue (Fig. 3D). To preliminarily explore the mechanism of how HFD upregulated the expression of CCL9, we conducted in vitro experiments to investigate the effects of fatty acids and adipocyte-CM on the expression of CCL9. Western blot and ELISA results revealed that adipocyte-CM significantly increased the expression of CCL9 in the SCC7 cell line, but the fatty acids PA and OA had no obvious effects (Supplementary Fig. S5A, B). This effect was further verified on primary oral epithelial cells from mice (Supplementary Fig. S5C), indicating that adipocytes may promote the expression of CCL9 in epithelial cells by secreting adipokines or other biomolecules instead of fatty acids.

To further evaluate the effect of CCL9 on MDSC migration, an in vitro chemotaxis assay was performed using a Transwell system. With the increased CCL9 content added to the medium in the bottom wells, the migration ability of MDSCs was enhanced (Fig. 3E), indicating that CCL9 possesses an effective MDSC chemotactic ability. Furthermore, we analyzed the expression of CCR1, the cognate receptor for CCL9 [23], in MDSCs from the upper and lower chambers after chemotaxis. The expression of CCR1 in MDSCs that migrated into the lower chamber increased significantly, while the expression of CCR1 in nonmigrated MDSCs in the upper chamber decreased (Fig. 3F), suggesting that CCL9 induces chemotaxis of MDSCs through CCR1. To further delineate chemotaxis by the CCL9/CCR1 axis in MDSC subpopulations, we next investigated whether M-MDSCs or PMN-MDSCs express CCR1. Flow cytometry analysis showed that PMN-MDSCs in the spleen, as well as in bone marrow, exhibited higher expression of CCR1, whereas M-MDSCs exhibited significantly lower expression of CCR1 (Fig. 3G). More importantly, the expression of CCR1 on PMN-MDSCs in the tongue of the HFD + 4NQO group was higher than that of the NFD + 4NQO group (Fig. 3H). Immunofluorescence analysis also showed that Gr1 (an MDSC marker) and CCR1 coexisted in dysplastic lesions, and the number of double-positive cells in the HFD + 4NQO group was higher than that in the NFD + 4NQO group (Fig. 3I). These results collectively indicated that MDSCs, mainly PMN-MDSCs, were recruited to the oral microenvironment through the CCL9/CCR1 axis in HFD mice.

### HFD enhanced the suppressive potency of MDSCs by increasing the intracellular uptake of lipids

**Fig. 4:**
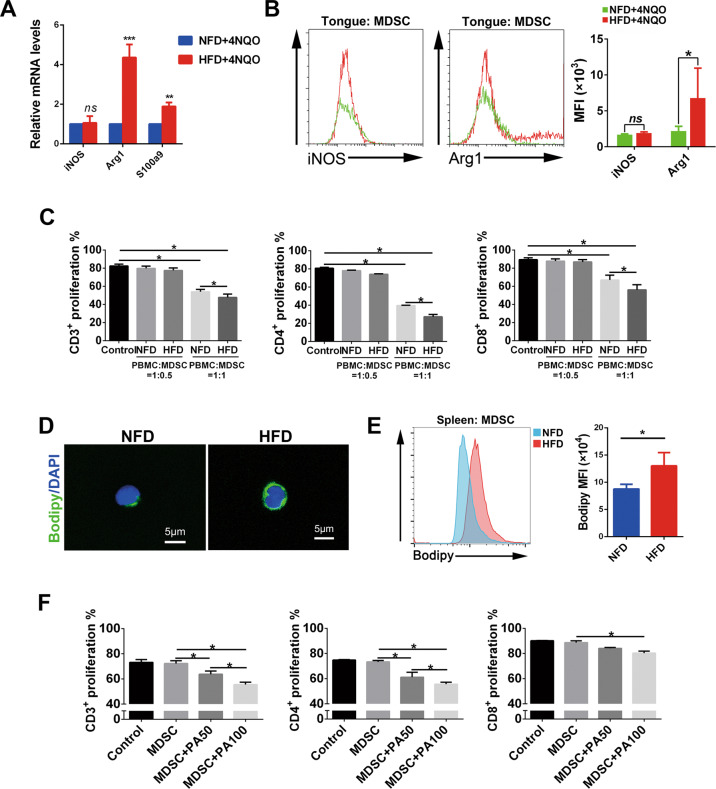
HFD enhanced the immunosuppressive potency of MDSCs by increasing the intracellular uptake of lipids. **A** The expression of immunosuppressive factors in the tongues of NFD + 4NQO and HFD + 4NQO mice was detected by qPCR. **B** The expression of immune factors in MDSCs from the tongues of NFD + 4NQO and HFD + 4NQO mice was detected by flow cytometry. **C** Quantification of the T cell proliferation assay. CellTrace™ violet fluorescence-labeled PBMCs were cocultured with MDSCs derived from NFD- or HFD-fed mice at ratios of 1:0, 1:0.5, or 1:1 and stimulated with anti-CD3/28 Dynabeads. After 3 days, cells were collected and stained with anti-mouse mAbs against CD3, CD4, and CD8 and further quantified using flow cytometry. **D** Representative confocal images of BODIPY 493/503 staining to detect intracellular lipids of MDSCs from NFD or HFD mice. Cell nuclei were counterstained with DAPI. **E** Flow cytometry assay showed the intracellular lipids of MDSCs from NFD or HFD mice. **F** T cell proliferation assays revealed that MDSCs pretreated with PA (50 μM or 100 μM) displayed enhanced suppression of CD3^+^, CD4^+^, and CD8^+^ T cells when cocultured with PBMCs at a ratio of 1:0.5. single asterisk (*) indicates *P* < 0.05, double asterisks (**) indicate *P* < 0.01, triple asterisks (***) indicate *P* < 0.001. Unpaired *t*-test; one-way ANOVA. NFD normal-fat diet, HFD high-fat diet, PBMCs peripheral blood mononuclear cells, MDSCs myeloid-derived suppressor cells, PA palmitic acid, PA50 PA at a concentration of 50 μM, PA100 PA at a concentration of 100 μM.

In addition to the recruitment of MDSCs, we further investigated whether a HFD had an influence on the suppressive potency of MDSCs in the oral microenvironment. MDSC-associated immunosuppressive factors were detected by qPCR in dysplastic lesions. The results showed that *Arg1* and *S100a9* were upregulated in the HFD + 4NQO group compared with the NFD + 4NQO group (Fig. 4A). Moreover, a flow cytometry assay was adopted to analyze the suppressor factors in local MDSCs. MDSCs in the HFD + 4NQO group exhibited elevated expression of Arg1 (Fig. 4B). For functional experiments, MDSCs derived from HFD- or NFD-fed mice were adopted to study their effect on T cell proliferation. When the ratio of peripheral blood mononuclear cells (PBMCs) to MDSCs was 1:0.5, neither MDSCs from the HFD group nor from the NFD group exhibited a suppressive effect. When the ratio of PBMCs to MDSCs was 1:1, MDSCs significantly inhibited the proliferation of CD3+, CD4+, and CD8+ T cells, and the MDSCs from the HFD group exhibited increased suppressive potency compared with those from the NFD group (Fig. 4C, Supplementary Fig. S6). As lipid accumulation is one of the most prominent features of a HFD, we further investigated whether the enhanced suppressive potency of MDSCs from a HFD was associated with intracellular lipid uptake. The presence of lipid droplets in MDSCs was detected by BODIPY staining. Confocal analysis (Fig. 4D) and flow cytometry assays (Fig. 4E) revealed that MDSCs in the HFD group had elevated intracellular lipid contents. To further analyze the effect of fatty acids on the suppressive activity of MDSCs, PA (the representative of saturated fatty acid) and OA (the representative of monounsaturated fatty acid) were used to treat MDSCs. T cell proliferation assays showed that MDSCs pretreated with PA or OA exhibited enhanced suppressive activity (Fig. 4F, Supplementary Fig. S7). Together, these results suggested that a HFD enhanced the suppressive potency of MDSCs by increasing the intracellular uptake of lipids.

### MDSC depletion ameliorated HFD-promoted carcinogenesis in model mice

**Fig. 5:**
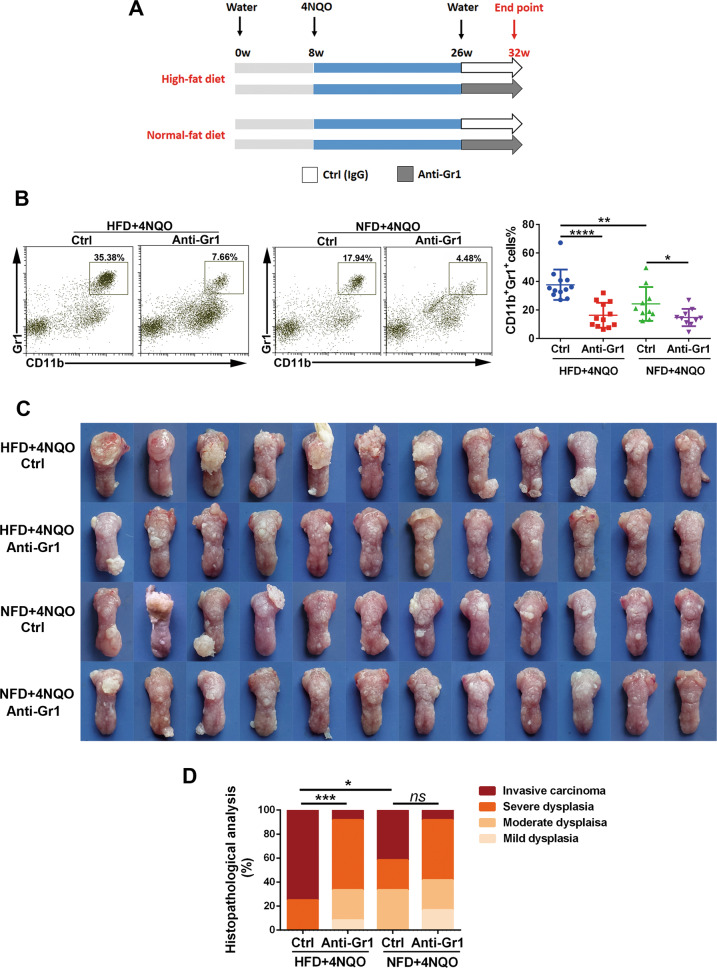
MDSC depletion significantly suppressed HFD-promoted carcinogenesis in model mice. **A** Schematic representation of the MDSC depletion experiment in the context of the diet-induced obesity model and experimental carcinogenesis. 4NQO concentration: 100 μg/mL. **B** Representative images of flow cytometry and quantification of CD11b^+^Gr1^+^ MDSCs in the tongue of mice after treatment. **C** Gross findings of lesions on the tongue of the model mice at the endpoint of the experiment. **D** Quantification of pathological grade in different groups. single asterisk (*) indicates *P* < 0.05, double asterisks (**) indicate *P* < 0.01, triple asterisks (***) indicate *P* < 0.001, quadruple asterisks (****) indicate *P* < 0.0001. One-way ANOVA; Kruskal–Wallis test. NFD normal-fat diet, HFD high-fat diet.

To assess the anticancer effect of targeting MDSCs in obesity, a well-characterized anti-Gr1 neutralizing monoclonal antibody (clone RB6-8C5) was used to deplete MDSCs in model mice (Fig. 5A). Mice treated with anti-Gr1 antibody in both diet groups had significantly decreased percentages of MDSCs in the blood, spleen, and tongue compared to those treated with the IgG control (Fig. 5B, Supplementary Fig. S8). Administration of anti-Gr1 antibody to NFD + 4NQO mice exhibited only minor inhibitory effects on the carcinogenesis process, whereas the treatment dramatically attenuated the severities of oral lesions in HFD + 4NQO mice (Fig. 5C). Histopathological evaluation showed severe epithelial dysplasia or invasive carcinoma in the HFD + 4NQO group, and the degree of malignancy was significantly reduced in the corresponding anti-Gr1 HFD + 4NQO group. However, there was no statistically significant difference in pathological grading between the anti-Gr1 antibody group and the IgG control group in NFD + 4NQO mice (Fig. 5D). These results suggested that depletion of MDSCs is effective at suppressing HFD-promoted carcinogenesis.

### Local adipocyte and CD33^+^ MDSC expansion was associated with poor survival in OSCC patients

**Fig. 6:**
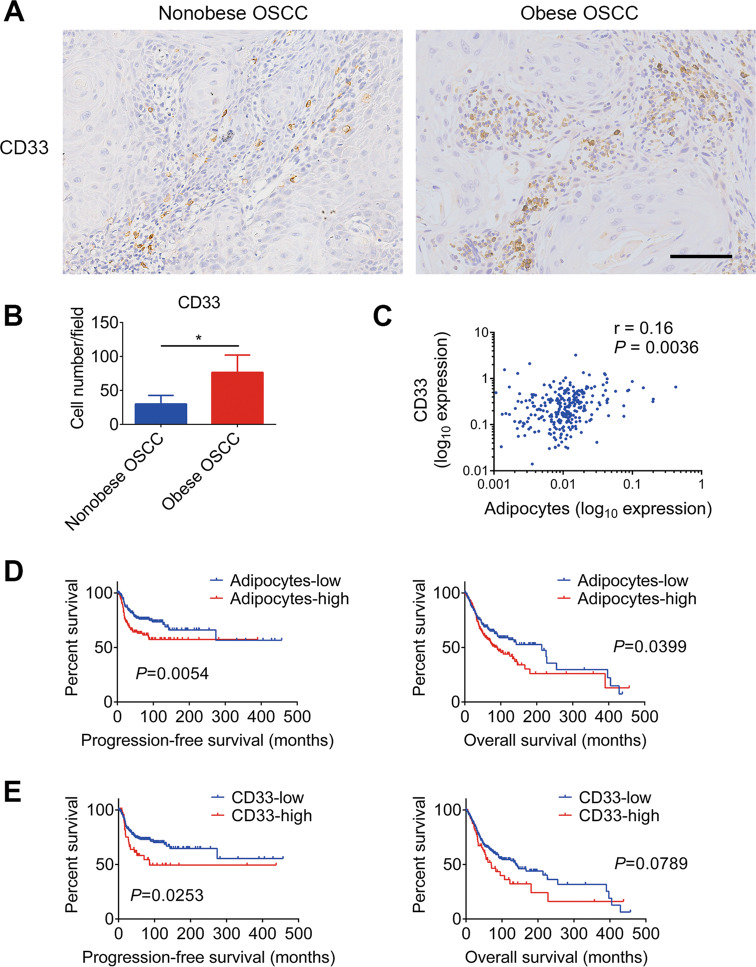
Local adipocyte and CD33^+^ MDSC expansion was associated with poor survival in OSCC patients. **A**, **B** Representative images and quantification of CD33 staining of OSCC specimens from nonobese (18.5 kg/m^2^ < BMI < 28 kg/m^2^) and obese (BMI ≥ 28 kg/m^2^) patients. **C** Pearson regression analysis revealed a positive correlation between local adipocyte abundance and CD33 expression in TCGA samples. **D**, **E** Patients in the TCGA datasets were divided into adipocyte-low and adipocyte-high groups and CD33-low and CD33-high groups based on their expression. Progression-free survival (PFS) and overall survival (OS) were analyzed using the Kaplan–Meier method and log-rank test. Single asterisk (*) indicates *P* < 0.05. Unpaired *t*-test; Pearson regression. BMI body mass index, TCGA The Cancer Genome Atlas.

We next examined the relationship between obesity and MDSC expansion in clinical samples. Using immunostaining, we evaluated the expression of CD33, a marker of human MDSCs, in tumor tissues from obese OSCC patients and nonobese patients. The results showed that infiltrated CD33^+^ cells were increased in the obese group (Fig. 6A, B). As adipocyte increases were observed in the tongue in obese conditions (Supplementary Fig. S1H, I), we further analyzed the correlation between local adipocyte abundance and CD33 expression based on the datasets from TCGA and xCell. The results indicated that local adipocyte abundance was significantly correlated with CD33 expression (Fig. 6C). Moreover, survival analysis showed that enrichment of adipocytes and high expression of CD33 were associated with poor overall survival (OS) (Fig. 6D) and progression-free survival (PFS) (Fig. 6E) in OSCC patients.

## Discussion

As a steadily growing epidemic, obesity has been strongly correlated with the increased risk and aggressiveness of many types of carcinoma [3, 24, 25]. However, the link between obesity and squamous cell carcinoma is not well constructed. A pooled analysis of 20 cohort studies including 1,941,300 participants demonstrated a positive association between BMI and head and neck squamous cell carcinoma (HNSCC) but only in nonsmokers [26], while central obesity (or abdominal obesity) measured by the waist-to-hip ratio (WHR) or waist circumference (WC) was linked with increased risk among both smokers and nonsmokers [27]. In our previous study, the prognosis of OSCC was poorer in obese patients than in normal-weight patients [4]. To further clarify the effect of dietary obesity on carcinogenesis in the head and neck, we used a 4NQO-induced mouse model in this study, which simulated the process of epithelial malignant transformation. Our results showed that a HFD promoted the initiation and development of OSCC, which provides evidence that diet-induced obesity was positively associated with the risk of squamous cell carcinoma.

Although obesity is a leading contributing factor to cancer risk, the mechanism by which it drives carcinogenesis is poorly understood. The emerging biological mechanisms linking obesity to cancer initiation include alterations in adipokine pathophysiology [28], abnormalities in the IGF-I axis and insulin resistance [29, 30], metabolic reprogramming caused by lipid uptake [31], the existence of subclinical chronic low-grade inflammation and oxidative stress [32], and changes in the local immune microenvironment [8, 33]. The poor survival of tongue squamous cell carcinoma in obese patients was reported to be correlated with white adipose tissue (WAT) locally [34]. Adipose is an active endocrine and immunological organ that secretes various types of adipokines, including leptin, adiponectin, resistin, and other cytokines, to modulate inflammation and regulate the local immune microenvironment [35, 36]. Therefore, we speculated that the local immune microenvironment of obese individuals may be altered and play important roles in the occurrence and development of OSCC. To characterize the local immune microenvironment, flow cytometry, immunofluorescence, and immunohistochemical experiments were performed in this study. Our results showed that CD11b^+^Gr1^+^ MDSCs were expanded in the tongue of HFD mice and that CD33^+^ MDSCs were increased in OSCC samples of obese patients.

The accumulation of MDSCs in the inflammatory or tumor microenvironment is regulated by a variety of factors, among which chemokines play an important role; chemokines can recruit MDSCs from the peripheral circulation to local tissues and promote the occurrence and development of tumors [37, 38]. In this study, we found that MDSCs, especially PMN-MDSCs, were increased in HFD mice and that CCL9 was highly expressed in epithelial cells. Furthermore, in vitro migration assays and in vivo analysis results confirmed that CCL9 promotes cell migration by acting on the CCR1 receptor of MDSCs. These results indicated that the CCL9/CCR1 axis may be a potential therapeutic target in obesity model mice. The MDSC-induced local immunosuppressive microenvironment is not only associated with quantity expansion but is also closely related to function. As obesity is a state of lipid accumulation, we investigated the effect of a HFD and fatty acid uptake on the function of MDSCs. Our data revealed that MDSCs from HFD-fed mice exhibited a stronger immunosuppressive function, which may be attributed to increased lipid uptake. Moreover, survival analysis showed that high expression of CD33, a human MDSC marker, was associated with poor prognosis. Thus, the alteration of MDSC quantity and activity in the oral microenvironment under HFD conditions indicated that targeting MDSCs may be an effective means to inhibit HFD-promoted tumorigenesis.

In the MDSC clearance experiments, we found that depletion of MDSCs effectively inhibited tumor formation in HFD mice. Notably, although depletion of MDSCs also reduced the malignant degree of lesions in NFD mice, the results did not show a significant difference, indicating that the treatment was more effective in obese conditions. This observation was consistent with recent reports, which suggested that immunotherapy was more effective in obese patients. A retrospective study found that obese patients with metastatic melanoma who received anti-PD-1/PDL-1 therapy had better PFS and OS than normal-weight patients [39]. Another study validated the results and extended the clinical data to a variety of cancer types including lung, melanoma, ovarian, and others. Their findings also proposed a mechanism for this phenomenon in which the improved outcomes may be due to obesity-induced T cell dysfunction resulting in higher PD-1 expression [40]. Our results provide new evidence that MDSCs may also be involved in the improved effectiveness of immunotherapy in obese patients.

To the best of our knowledge, a variety of research exploring the role of obesity on cancers particularly those adenocarcinomas, such as breast cancer, prostate cancer, and esophagus adenocarcinoma [3]. As for squamous cell carcinoma, we found the implication of obesity in OSCC has common and unique features compared with other tumors. Commonly, obesity promotes the initiation and development of cancers including OSCC, with dramatic disturbance of the immune microenvironment [6]. However, the relationship between obesity and OSCC has its unique characteristics. First, it is greatly affected by risk factors such as smoking and drinking [4, 26, 27]. If the influence of these interfering factors is not ruled out, conflicting conclusions may be drawn. Besides, we found that obesity interferes with the immune microenvironment of OSCC by recruiting MDSCs via the CCL9-CCR1 axis, highlighting the potential therapeutic value of targeting MDSCs and blocking CCL9-CCR1 axis in this context.

**Fig. 7:**
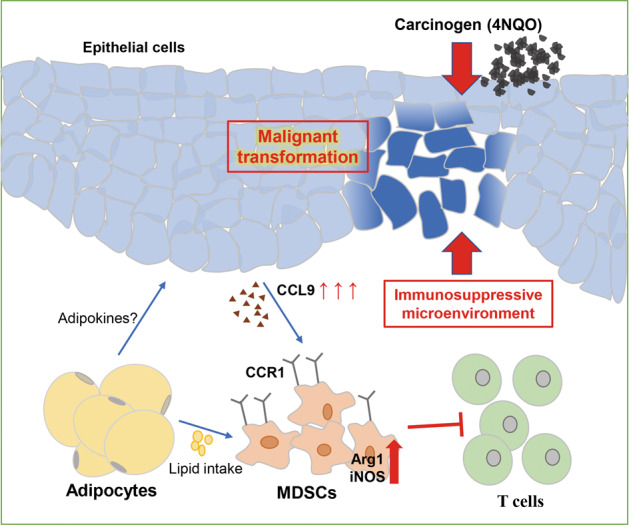
Schematic diagram. Schematic representation of the proposed mechanism of how obesity accelerates oral carcinogenesis by recruiting MDSCs and enhancing their immunosuppressive activities.

Collectively, our study has provided strong evidence that obesity is linked with the initiation and progression of OSCC. The underlying mechanism may be to induce an immunosuppressive microenvironment by recruiting MDSCs through the CCL9/CCR1 axis and by functionally enhancing MDSCs via intracellular fatty acid uptake (Fig. 7). Remarkably, depletion of MDSCs significantly ameliorated HFD-promoted carcinogenesis in model mice but showed a weak effect in NFD mice. Our findings advance the understanding of obesity-mediated immune dysfunction and its role in cancer progression and emphasize the importance of cancer immunotherapy in obese patients.

## Supplementary information

Supplementary Figure Legends

Supplementary Fig. S1

Supplementary Fig. S2

Supplementary Fig. S3

Supplementary Fig. S4

Supplementary Fig. S5

Supplementary Fig. S6

Supplementary Fig. S7

Supplementary Fig. S8

## Data Availability

The datasets used and/or analyzed during the current study are available from the corresponding author on reasonable request.
