# Supplementary material for: Diet-induced obesity accelerates oral carcinogenesis by recruitment and functional enhancement of myeloid-derived suppressor cells
Source: Cell Death Dis. 2021 Oct 14;12(10):946. doi: 10.1038/s41419-021-04217-2 (PMC8516872; doi:10.1038/s41419-021-04217-2)
Supplement: Supplementary file 1 — Supplementary Figure Legends [file 41419_2021_4217_MOESM1_ESM.docx]

**Supplementary Figure Legends**

**Supplementary Fig. S1 (A)** Representative images of the general condition of the model mice at week 24. **(B)** The body weight curves of the model mice over the experiment. **(C-E)** Changes in fasting blood glucose **(C)**, serum TC **(D)**, and serum TG **(E)** of the model mice during the experiment. **(F-G)** After 24 weeks of feeding on a high-fat diet, fatty liver appeared. Representative images and quantification of oil red O staining of the liver are shown. **(H-I)** H&E staining was used to observe and quantitatively measure the number and size of adipocytes in the tongues of the model mice. * indicates *P* < 0.05, ** indicates *P* < 0.01, *** indicates *P* < 0.001, **** indicates *P* < 0.0001. Unpaired t-test. NFD, normal-fat diet; HFD, high-fat diet; TC, total cholesterol; TG, triglyceride; ORO, oil red O.

**Supplementary Fig. S2 (A)** After 16 weeks of 4NQO induction (end point 1), a few spot-like white patches appeared on the tongue dorsum of mice. **(B)** The pathological manifestations were dysplasia with an intact epithelial basement membrane. The infiltration of immune cells increased in the lesions of the HFD+4NQO group. NFD, normal-fat diet; HFD, high-fat diet.

**Supplementary Fig. S3** Gating strategy for the immune cell population analysis in the tongue.

**Supplementary Fig. S4 (A-B)** Representative dot plot and quantification of CD11b^+^Gr1^+^ MDSCs in the spleen or blood of NFD+4NQO and HFD+4NQO mice by flow cytometry. **(C-D)** Representative dot plot and quantification of CD11b^+^Gr1^+^ MDSCs in the spleen or blood of NFD and HFD mice by flow cytometry (HFD feeding for 8 weeks without 4NQO exposure). * indicates *P* < 0.05. Unpaired t-test. NFD, normal-fat diet; HFD, high-fat diet.

**Supplementary Fig. S5 (A)** Western blot results revealed that the expression of CCL9 in the SCC7 cell line significantly increased after treatment with adipocyte-CM for 48 h, but there was no obvious effect with the fatty acid PA (50 μM or 100 μM) and OA (100 μM or 200 μM) treatment. **(B-C)** ELISA results verified that adipocyte-CM significantly increased the expression of CCL9 in both the SCC7 cell line and primary oral epithelial cells from mice. ** indicates *P* < 0.01, **** indicates *P* < 0.0001. Unpaired t-test. CM, conditioned media; PA, palmitic acid; PA50, PA at a concentration of 50 μM; PA100, PA at a concentration of 100 μM; OA, oleic acid; OA100, OA at a concentration of 100 μM; OA200, OA at a concentration of 200 μM.

**Supplementary Fig. S6** Representative flow cytometry diagrams of the T cell proliferation assay. CellTrace™ violet fluorescence-labeled PBMCs were cocultured with MDSCs derived from NFD- or HFD-fed mice at ratios of 1:0, 1:0.5, or 1:1 and stimulated with anti-CD3/28 Dynabeads. After 3 days, cells were collected and stained with anti-mouse mAbs against CD3, CD4, and CD8 and further quantified using flow cytometry. Quantification results are shown in Fig. 4C. NFD, normal-fat diet; HFD, high-fat diet; PBMCs, peripheral blood mononuclear cells; MDSCs, myeloid-derived suppressor cells.

**Supplementary Fig. S7** Flow cytometry analysis of fatty acids on the inhibitory activity of MDSCs. CellTrace™ violet fluorescence-labeled PBMCs were cocultured with MDSCs pretreated with PA **(A)** or OA **(B)** at a ratio of 1:0.5 and stimulated with anti-CD3/28 Dynabeads. After 3 days, cells were collected and stained with anti-mouse mAbs against CD3, CD4, and CD8 and further quantified using flow cytometry. * indicates *P* < 0.05. One-way ANOVA. PBMCs, peripheral blood mononuclear cells; MDSCs, myeloid-derived suppressor cells; PA, palmitic acid; OA, oleic acid; OA100, OA at a concentration of 100 μM; OA200, OA at a concentration of 200 μM.

**Supplementary Fig. S8** Representative images of flow cytometry and the quantification of CD11b^+^Gr1^+^ MDSCs in blood **(A)** and spleen **(B)** from the mice in the MDSC depletion experiment. * indicates *P* < 0.05, *** indicates *P* < 0.001, **** indicates *P* < 0.0001. One-way ANOVA. NFD, normal-fat diet; HFD, high-fat diet.
