# Supplementary figures and images for: Diet-induced obesity accelerates oral carcinogenesis by recruitment and functional enhancement of myeloid-derived suppressor cells
Source: Cell Death Dis. 2021 Oct 14;12(10):946. doi: 10.1038/s41419-021-04217-2 (PMC8516872; doi:10.1038/s41419-021-04217-2)

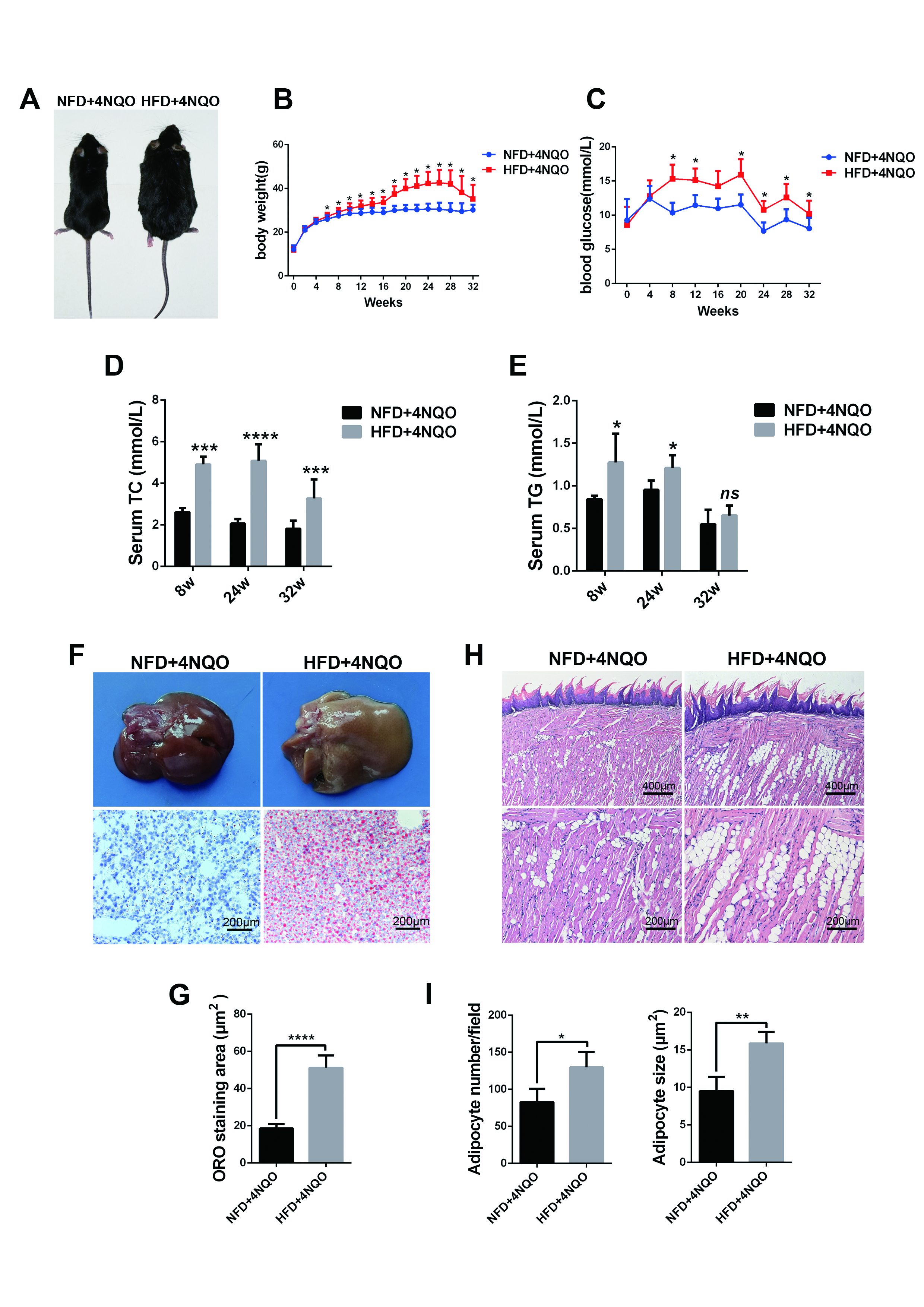

Supplement: Supplementary file 2 — Supplementary Fig. S1 [file 41419_2021_4217_MOESM2_ESM.tif]

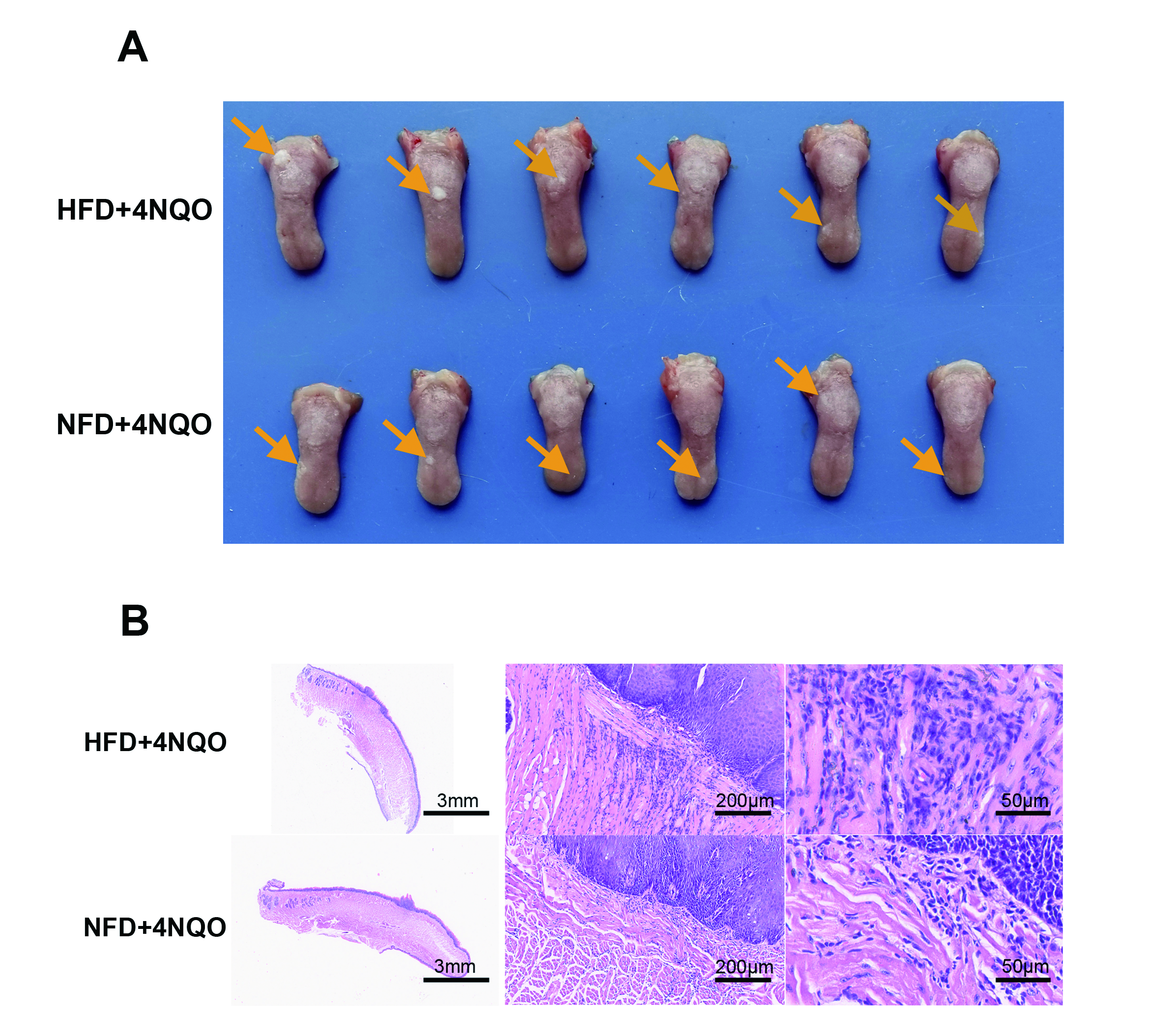

Supplement: Supplementary file 3 — Supplementary Fig. S2 [file 41419_2021_4217_MOESM3_ESM.tif]

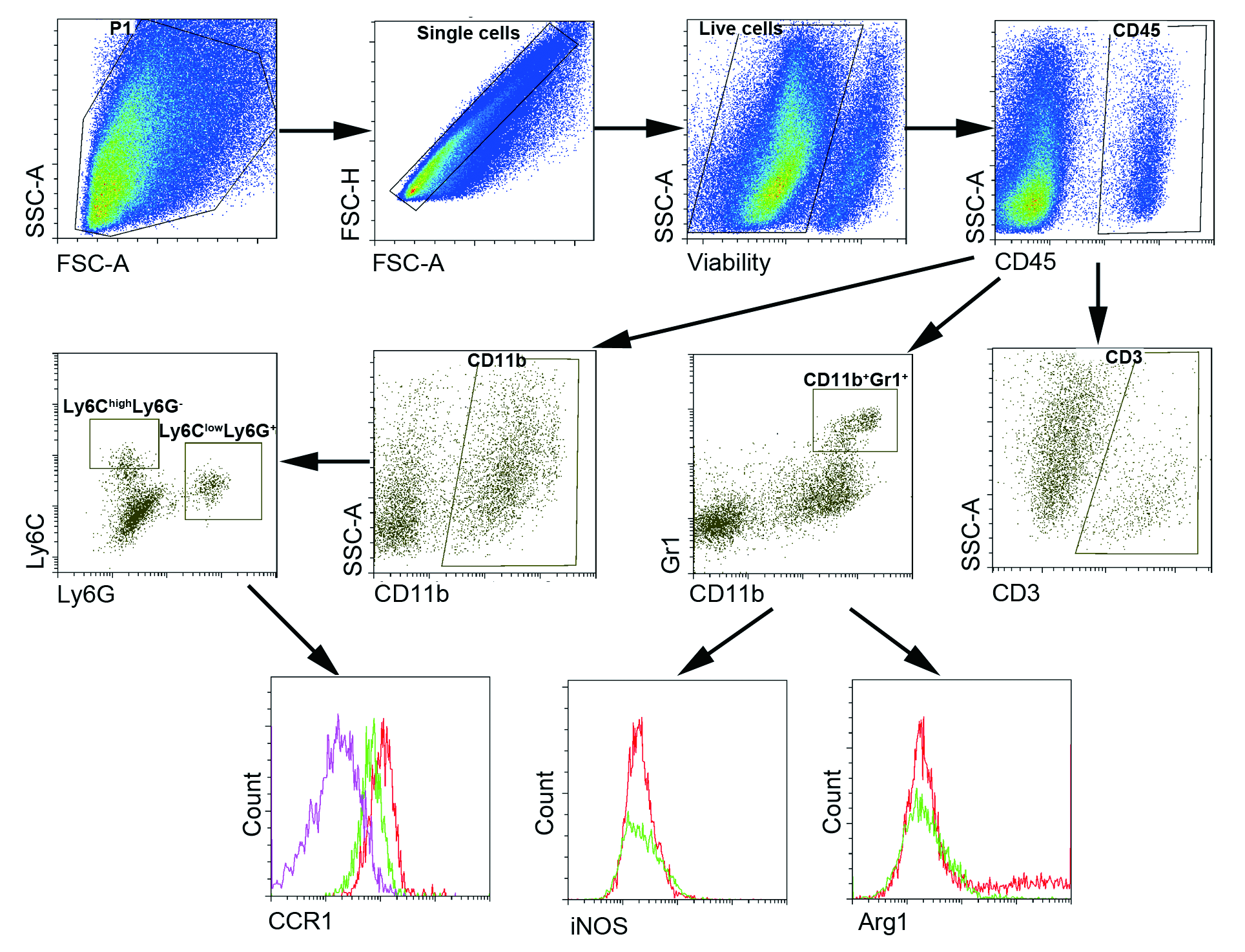

Supplement: Supplementary file 4 — Supplementary Fig. S3 [file 41419_2021_4217_MOESM4_ESM.tif]

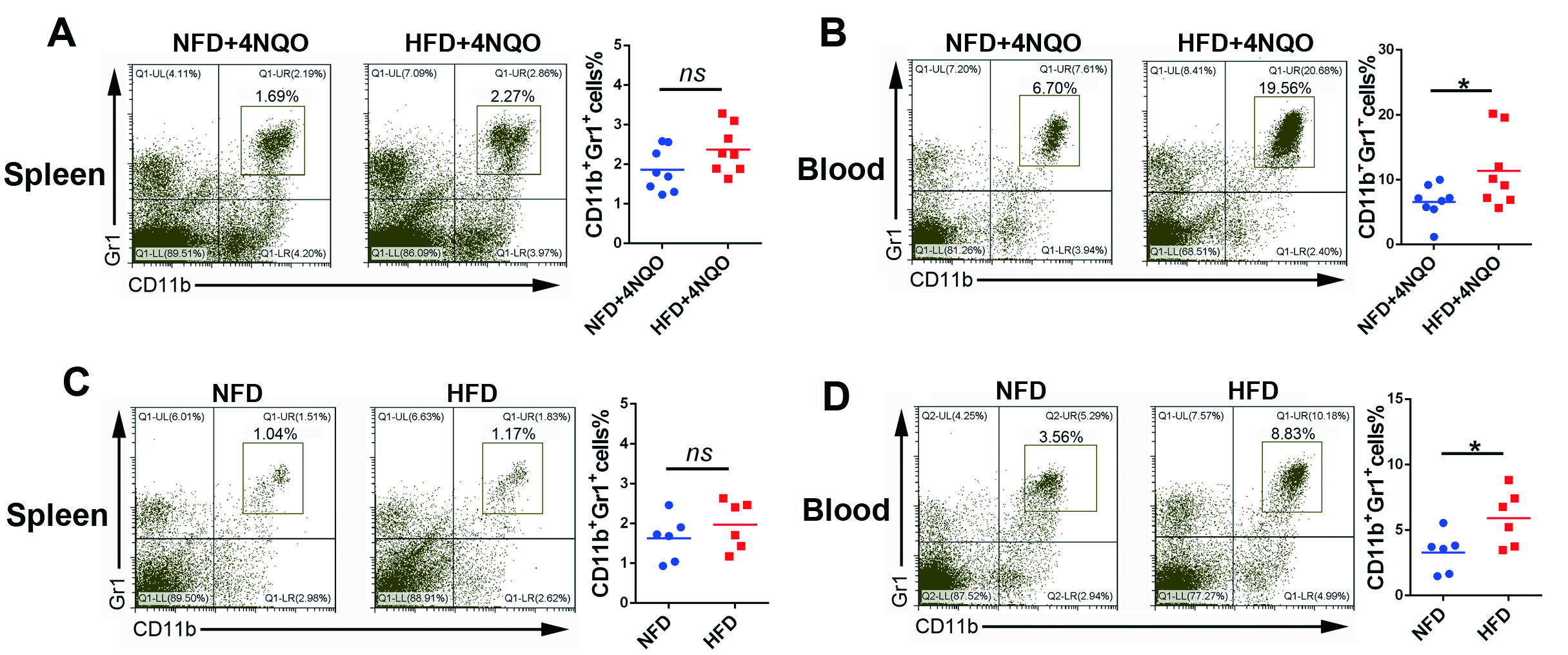

Supplement: Supplementary file 5 — Supplementary Fig. S4 [file 41419_2021_4217_MOESM5_ESM.tif]

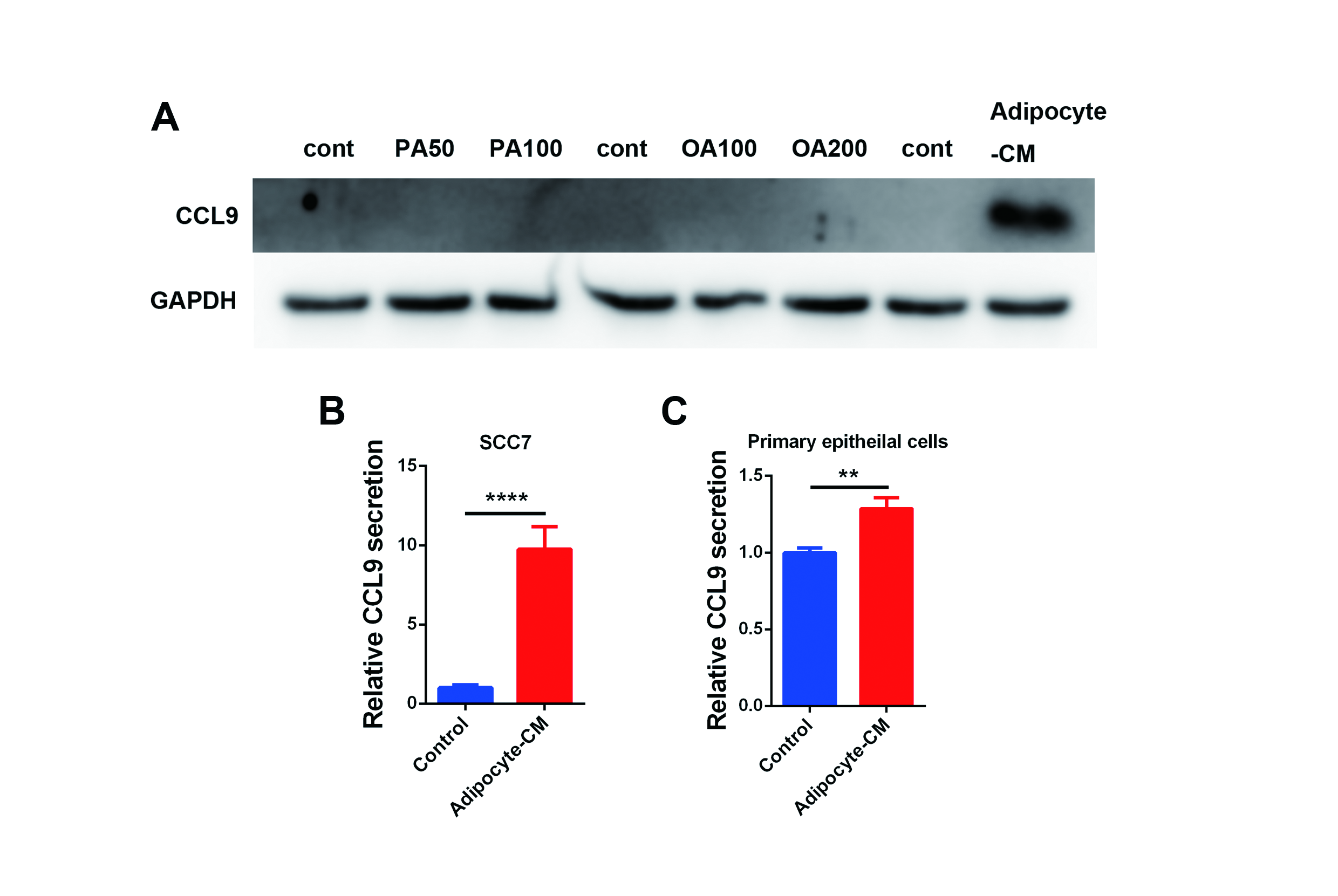

Supplement: Supplementary file 6 — Supplementary Fig. S5 [file 41419_2021_4217_MOESM6_ESM.tif]

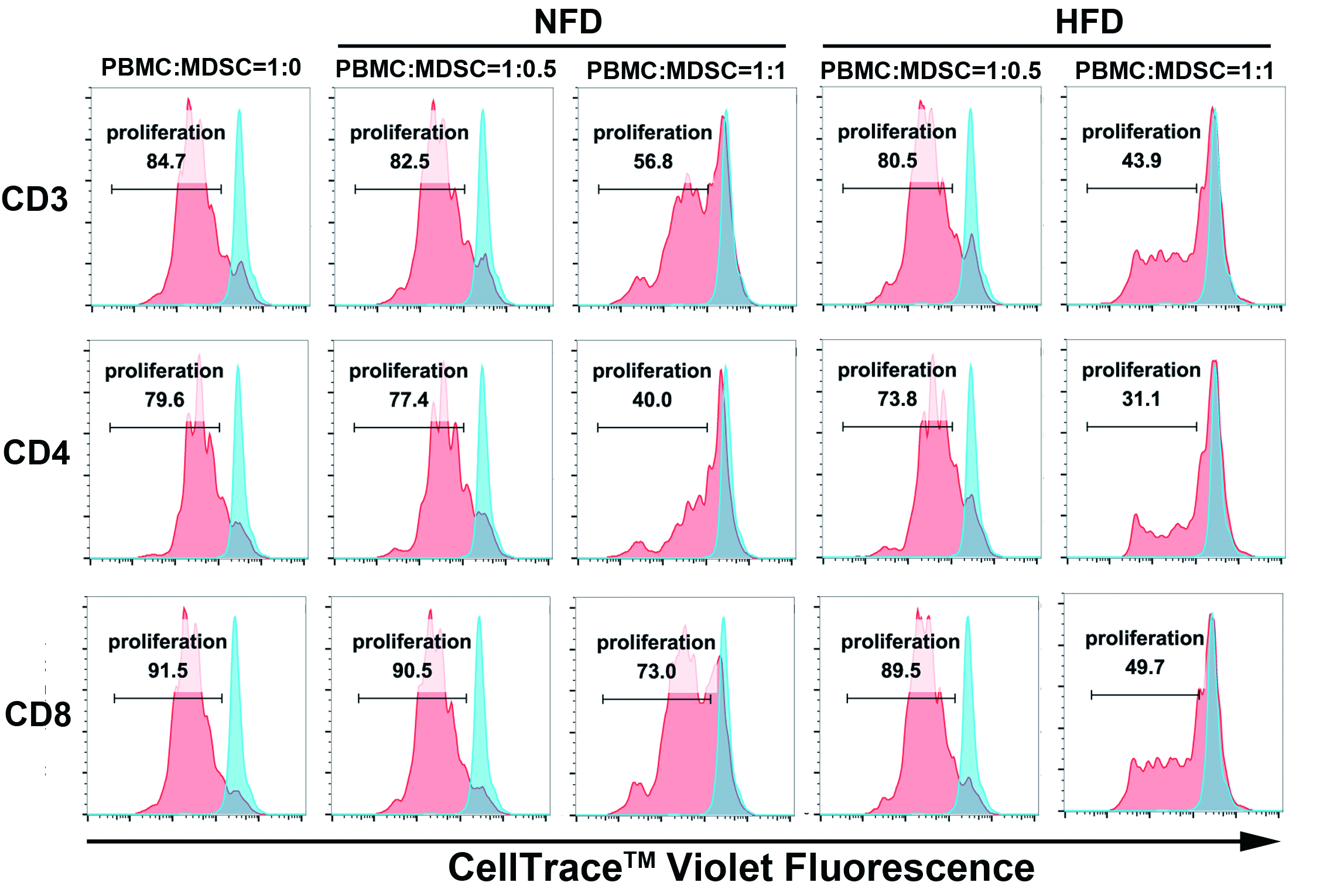

Supplement: Supplementary file 7 — Supplementary Fig. S6 [file 41419_2021_4217_MOESM7_ESM.tif]

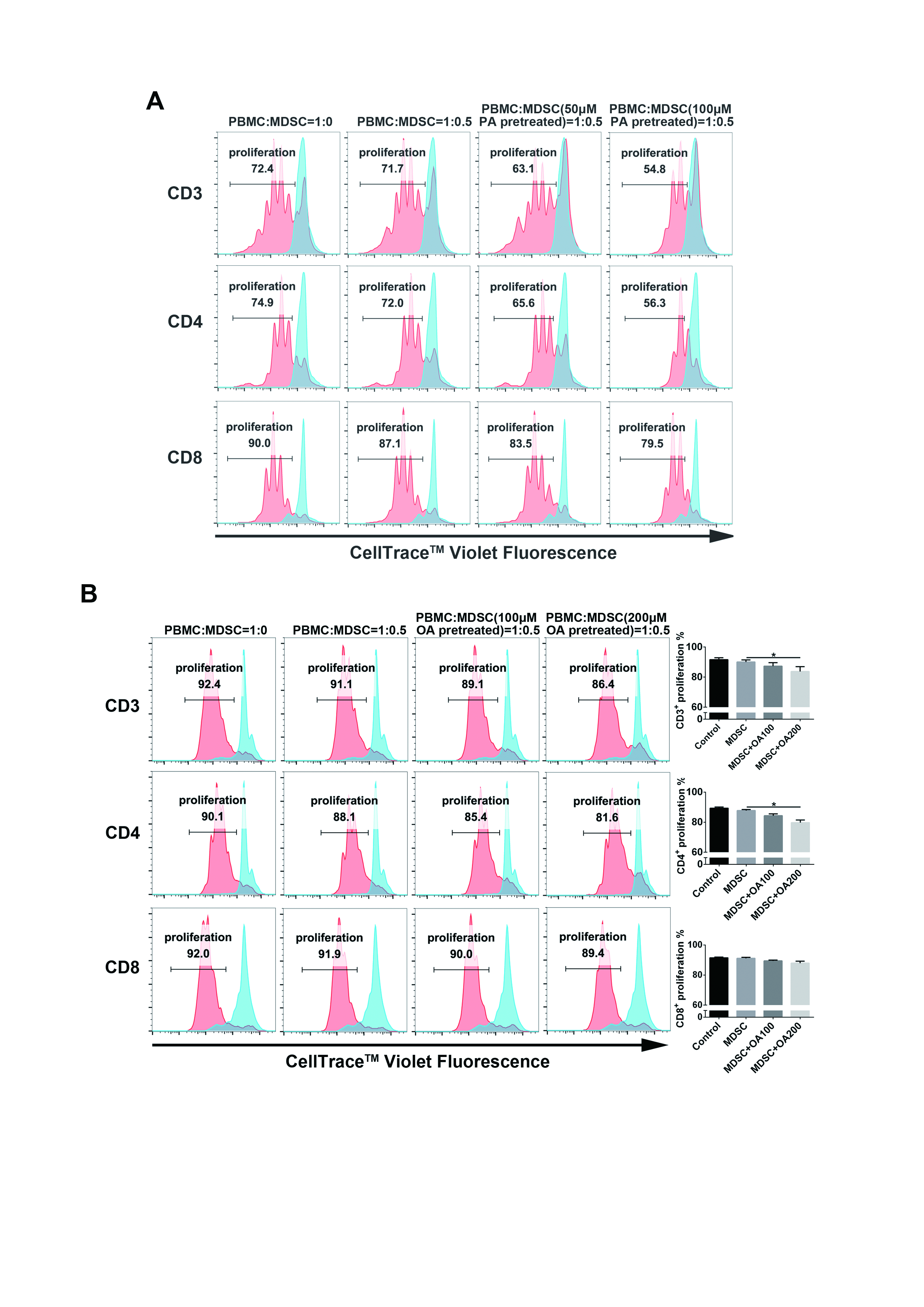

Supplement: Supplementary file 8 — Supplementary Fig. S7 [file 41419_2021_4217_MOESM8_ESM.tif]

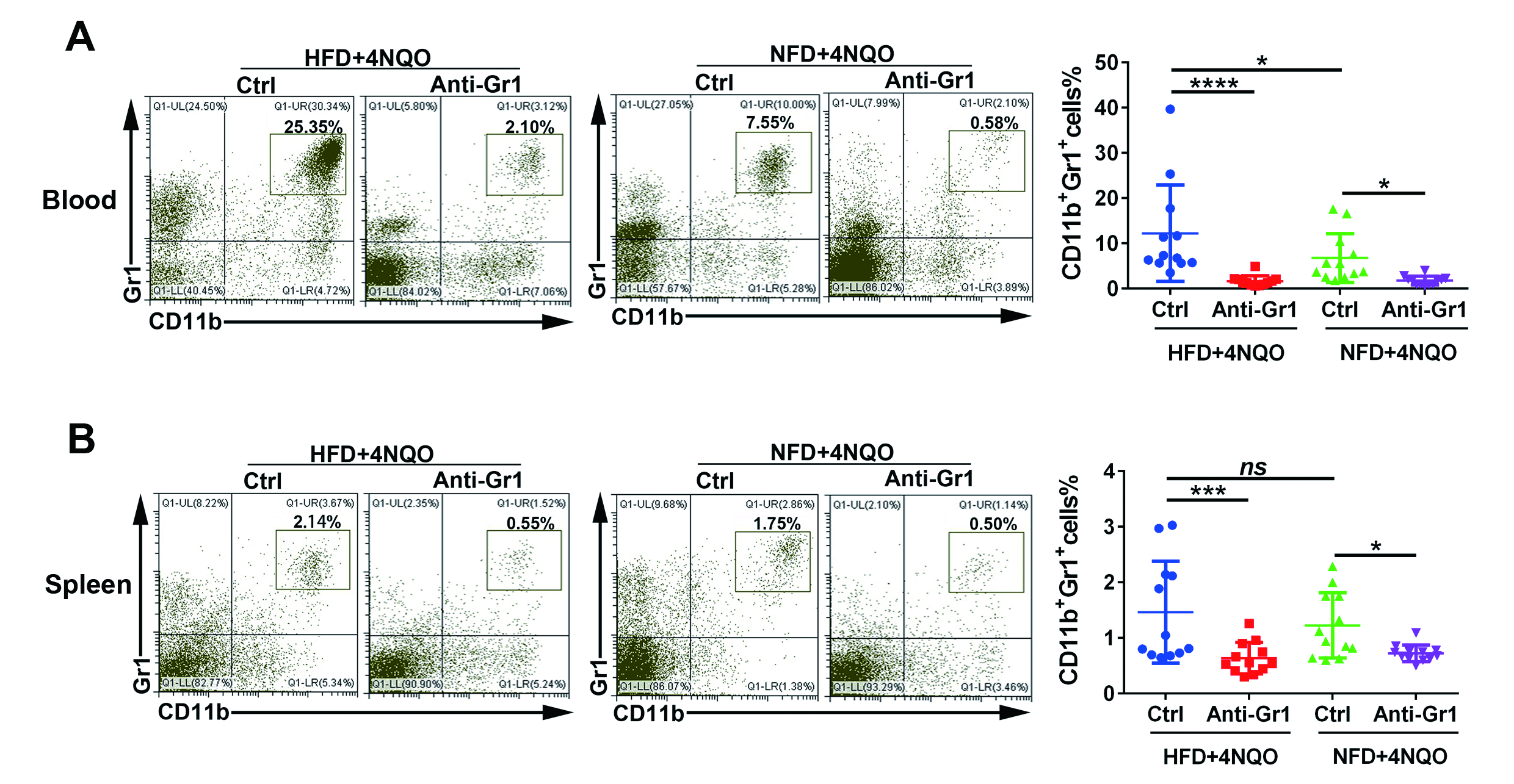

Supplement: Supplementary file 9 — Supplementary Fig. S8 [file 41419_2021_4217_MOESM9_ESM.tif]
